# Singlet-like correlations: equal peak work, unequal robustness

**DOI:** 10.1140/epjp/s13360-026-07804-4

**Published:** 2026-05-20

**Authors:** Karl Svozil

**Affiliations:** Institute for Theoretical Physics, TU Wien, Wiedner Hauptstrasse 8-10/136, 1040 Vienna, Austria

## Abstract

Initial system–environment correlations are a thermodynamic resource, enabling work extraction through their erasure. We compare three representative *singlet-like, rotationally covariant* correlation laws—a local classical benchmark, the quantum cosine law and an idealized stronger-than-quantum step law—under measurement misalignment. In the binary-outcome, uniform-marginal setting, all three can attain the same peak extractable work, $k_{\mathrm B}T\ln 2$. Their operational value differs, however, in robustness away from perfect alignment. For the chosen classical benchmark, the mutual information degrades as $\Theta (\delta \theta \ln (1/\delta \theta ))$, whereas for the quantum cosine law it degrades as $\Theta (\delta \theta ^2\ln (1/\delta \theta ))$. The stronger-than-quantum step law is perfectly flat except at a critical angle. Accordingly, the paper establishes a robustness hierarchy *within this restricted comparison class*, rather than a no-go theorem for all classical correlations.

## Introduction

Classical thermodynamics defines an isolated system by boundaries impermeable to matter and energy. This picture is challenged by microscopic systems prepared with initial correlations to their environment. Even when sealed against energy exchange, such a system’s local state can be altered by operations on its correlated environmental counterpart. An agent possessing side information can consume these correlations to perform work, a process governed by generalized thermodynamic laws that incorporate information-theoretic balances [1–3].

The connection between information and thermodynamics has a long history, originating with Maxwell’s “demon” thought experiment and formalized through the work of Szilard [4], who showed that a single bit of information about a molecule’s position in a partitioned box can be converted into $k_{\textrm{B}}T\ln 2$ of work. Landauer [5] later established that this cycle is closed by the erasure cost: Resetting the demon’s memory dissipates at least $k_{\textrm{B}}T\ln 2$ of heat, thereby saving the second law [6, 7].

In the quantum regime, information-thermodynamic trade-offs are enriched by genuinely nonclassical correlations. It has been shown that quantum discord [8, 9] and quantum entanglement [2] can serve as thermodynamic resources, and that the framework of generalized free energies [10–12] extends the second law to the single-shot regime. Sagawa and Ueda [13, 14] established rigorous equalities connecting feedback control, measurement and work extraction in the quantum setting. Comprehensive reviews of the field can be found in Refs. [15, 16].

This paper compares three representative correlation laws and shows that, within a natural singlet-like comparison class, their peak energetic value can coincide while their robustness against misalignment differs sharply. For the three representative singlet-like laws studied here, greater nonlocality coincides with greater thermodynamic robustness under misalignment. This robustness hierarchy is quantified by an aggregate thermodynamic parameter inspired by the measurement settings of the Clauser–Horne–Shimony–Holt (CHSH) scenario [17–19], though this parameter is used as an operational figure of merit rather than as a Bell inequality.

The remainder of this paper is organized as follows. Section 2 reviews the information-theoretic framework connecting mutual information to extractable work and derives the mutual information for binary-outcome correlations. Section 3 describes the operational work extraction protocol. Section 4 introduces the three representative correlation laws. Section 5 analyzes their thermodynamic value and robustness, including a Bell inequality argument that constrains the small-angle behavior of local singlet-like models. Section 6 defines and evaluates an aggregate thermodynamic robustness parameter across multiple measurement settings. Section 7 discusses extensions and limitations. Section 8 positions the work relative to the existing literature. Section 9 concludes.

## Preliminaries

### From information to work

Consider a bipartite system consisting of a subsystem *A* and its environment *E*, jointly described by a state $\rho _{AE}$ with reduced states $\rho _A = \textrm{Tr}_E[\rho _{AE}]$ and $\rho _E = \textrm{Tr}_A[\rho _{AE}]$. The quantum mutual information,

1 $$\begin{aligned} I(A:E) = S(\rho _A) + S(\rho _E) - S(\rho _{AE}), \end{aligned}$$

where $S(\rho ) = -\textrm{Tr}[\rho \ln \rho ]$ is the von Neumann entropy, quantifies the total correlations (both classical and quantum) between *A* and *E*.

If *A* is in contact with a thermal bath at temperature *T*, and the agent performs a cyclic process on *A* that begins with correlations $I_i(A{:}E)$ and terminates in an uncorrelated product state $\rho _A\otimes \rho _E$, the generalized second law [1] imposes the following bound on the extractable work:

2 $$\begin{aligned} W_{\textrm{ext}} \le -\Delta F_T(\rho _A) + k_{\textrm{B}} T\, I_i(A{:}E), \end{aligned}$$

where $\Delta F_T(\rho _A)$ is the change in the nonequilibrium free energy of subsystem *A*. This result can be understood intuitively: The mutual information $I_i(A{:}E)$ represents a bonus resource, additional to the system’s own free energy, that an informed agent can exploit. When the process is cyclic on *A*, so that $\Delta F_T(\rho _A)=0$, this simplifies to

3 $$\begin{aligned} W_{\textrm{ext}} \le k_{\textrm{B}} T\, I_i(A{:}E). \end{aligned}$$

The physical content of Eq. (3) generalizes Szilard’s result [4]: One bit of mutual information ($I = \ln 2$ nats) can be reversibly converted into $k_{\textrm{B}}T\ln 2$ of work, precisely the energy liberated by an isothermal expansion of a single-molecule gas when the piston’s initial position is known. Following del Rio et al. [2], the work cost of erasing a system is governed by the conditional entropy $S(A|E) = S(\rho _{AE}) - S(\rho _E)$, which can be negative for entangled states, permitting work *gain* during erasure. The work bound in Eq. (3) is consistent with this picture, since for a maximally mixed marginal $\rho _A$ (entropy $S(\rho _A) = \ln 2$), we have $I(A{:}E) = \ln 2 - S(A|E)$.

*Scope of the bound.* The quantum mutual information of Eq. (1) can exceed $\ln 2$ for quantum subsystems (for instance, $I(A{:}E) = 2\ln 2$ for a maximally entangled two-qubit state), and in principle, more general protocols could exploit such larger correlations [2]. In the present work, we restrict attention to the *outcome-level* mutual information arising from binary measurements with uniform marginals, for which $I \le \ln 2$ always holds. The bound $W \le k_{\textrm{B}}T \ln 2$ is therefore specific to this one-unbiased-bit operational setting, not universal across all quantum thermodynamic protocols.

### Mutual information from correlations

We now specialize to a scenario directly relevant to Bell-type experiments. Consider two parties sharing a no-signaling bipartite system with binary outcomes $a, e \in \{+1, -1\}$ and uniform marginals: $P(a) = P(e) = 1/2$. In this case, the joint distribution is entirely determined by the correlation function

4 $$\begin{aligned} {E(\theta ) = \langle a\, e \rangle _\theta = \sum _{a,e} a\, e\, P(a,e|\theta ),} \end{aligned}$$

where $\theta$ is the relative angle between the two measurement settings. Explicitly, the joint probabilities are

5 $$\begin{aligned} P(a = e|\theta ) = \frac{1+E(\theta )}{2}, \qquad P(a \ne e|\theta ) = \frac{1-E(\theta )}{2}. \end{aligned}$$

The marginal entropy is $H(A) = \ln 2$. The conditional entropy is the binary entropy of the error probability,

6 $$\begin{aligned} H(A|E) = h_2\!\left( \frac{1-E(\theta )}{2}\right) = h_2\!\left( \frac{1+E(\theta )}{2}\right) , \end{aligned}$$

where $h_2(p) = -p\ln p - (1{-}p)\ln (1{-}p)$ is the binary Shannon entropy in nats, and the last equality follows from the symmetry $h_2(p)=h_2(1{-}p)$. The mutual information is therefore

7 $$\begin{aligned} I_\theta (A{:}E) = {H(A) - H(A|E) =} \ln 2 - h_2\!\left( \frac{1+E(\theta )}{2}\right) . \end{aligned}$$

This result depends on the correlation $E(\theta )$ only through its absolute value $|E(\theta )|$, via the binary entropy. Perfect correlation or anti-correlation ($|E|=1$) gives $I = \ln 2$; zero correlation ($E=0$) gives $I = 0$. Equation (7) is model-independent: It holds for any bipartite device (or theory) whose outputs are binary with uniform marginals, in which case the joint distribution is fully determined by the correlator $E(\theta )=\langle AE\rangle _\theta$.

## Operational protocol

To make the work extraction scenario concrete, we describe an operational protocol inspired by the Szilard engine [4, 13]. We treat the measurement outcomes as classical registers; hence, $I_\theta (A{:}E)$ denotes the Shannon mutual information of the binary variables $A,E\in \{\pm 1\}$.

1. Alice and Bob share a correlated resource (classical shared randomness, an entangled quantum state or a no-signaling box).
2. A referee assigns measurement settings: Alice receives setting *x* (direction $\alpha _x$) and Bob receives setting *y* (direction $\beta _y$). The settings (equivalently, the relative angle $\theta =\alpha _x-\beta _y$) are assumed to be known to both parties (e.g., publicly announced by the referee).
3. Alice performs her measurement and obtains $a\in \{+1,-1\}$. Bob queries his device with setting *y*, obtaining $e\in \{+1,-1\}$, which is stored in a physical two-state working medium (e.g., Left/Right of a one-molecule Szilard box at temperature *T*).
4. Alice communicates her one-bit result *a* to Bob via a classical channel.
5. Using *a* and the known correlator $E(\theta )=\langle AE\rangle _\theta$, Bob performs a reversible relabeling (a conditional flip) of his two-state working medium and defines an agreement variable
  $$ z:= \sigma (\theta )\, a e \in \{+1,-1\}, $$
  where $\sigma (\theta )\in \{+1,-1\}$ is chosen to maximize $P(z=+1|\theta )$ (equivalently, $\sigma (\theta )=\textrm{sgn}\,E(\theta )$ whenever $E(\theta )\ne 0$). For uniform marginals,
  8 $$\begin{aligned} {\begin{matrix} p_{\textrm{corr}}(\theta ) & = P(z=+1|\theta ) \\ & = \max \!\Big \{\frac{1+E(\theta )}{2},\frac{1-E(\theta )}{2}\Big \} \\ & = \frac{1+|E(\theta )|}{2}\,. \end{matrix}} \end{aligned}$$
6. Bob now performs a feedback-controlled quasi-static isothermal protocol that brings his two-state working medium from the biased distribution *P*(*z*) to thermal equilibrium (uniform). The *A*–*E* correlation is the informational resource being consumed. The maximum average extractable work per run is [13, 20]
  9 $$\begin{aligned} W(\theta ) = k_{\textrm{B}}T\,I_\theta (A{:}E) = k_{\textrm{B}}T\!\left[ \ln 2 - h_2\!\left( \frac{1+|E(\theta )|}{2}\right) \right] \!. \end{aligned}$$

Since Alice communicates exactly one classical bit and the marginals are uniform, the mutual information satisfies $I_\theta (A{:}E) \le H(A) = \ln 2$; hence, $W(\theta )\le k_{\textrm{B}}T\ln 2$ in this one-unbiased-bit setting [5, 6]. This bound is saturated when $|E(\theta )|=1$, i.e., when Bob can deterministically predict (equivalently, deterministically relabel) the logical state of his two-state working medium.

*Thermodynamic bookkeeping.* Equation (9) quantifies the *gross* work that can be extracted from Bob’s two-state working medium given access to Alice’s one-bit side information. The energetic cost of creating, transmitting, storing and eventually resetting the communication record is not included in $W(\theta )$. Accordingly, our analysis compares the conditional work value of different correlation laws under a common one-bit feedforward resource; it does not claim a positive *net* work gain for a fully closed engine cycle once communication and memory reset costs are included.

## A hierarchy of correlation laws

Our aim is not to classify all local hidden-variable correlations. Without further restrictions, trivial local strategies with unbiased marginals can produce angle-independent perfect anti-correlation, e.g., $E(\theta )\equiv -1$, and therefore a flat work profile. Such strategies do not model a singlet-like angular response and are not part of the present comparison. Throughout, we restrict attention to singlet-like, angle-dependent, rotationally covariant correlation laws with unbiased marginals satisfying

10 $$\begin{aligned} E(0)=-1,\qquad E(\pi )=+1,\qquad E(\pi -\theta )=-E(\theta ), \end{aligned}$$

together with monotonic increase on $[0,\pi ]$. Within this restricted class, the linear local model serves as a classical benchmark.

**Fig. 1:**
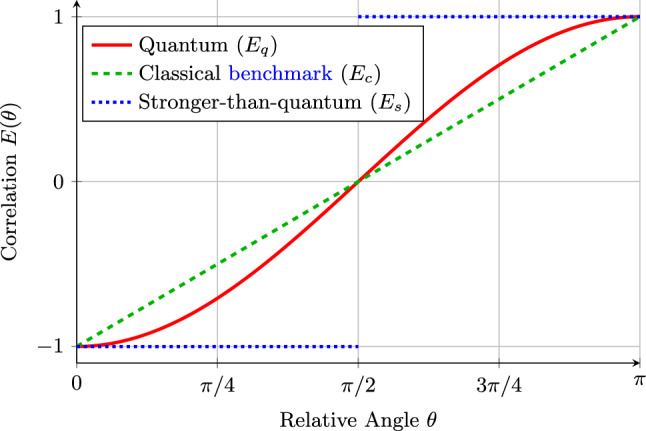
Comparison of the three representative correlation functions $E(\theta )$. The quantum (solid red) and classical benchmark (dashed green) only achieve perfect anti-correlation at $\theta =0$. The stronger-than-quantum correlation (dotted blue) is perfect for all angles except $\theta = \pi /2$

We analyze three representative bipartite correlation laws $E(\theta )$ for $\{\pm 1\}$-outcome measurements as a function of the relative angle $\theta \in [0, \pi ]$ between settings. Their nonlocality is gauged by the CHSH parameter [17, 18]

11 $$\begin{aligned} \mathcal {S}_{\textrm{CHSH}} {= |E(\theta _1,\theta _2) + E(\theta _1,\theta _2') + E(\theta _1',\theta _2) - E(\theta _1',\theta _2')|,} \end{aligned}$$

for which local realism imposes a bound of 2, quantum mechanics a bound of $2\sqrt{2}$ (the Tsirelson bound [21]) and the no-signaling principle an algebraic limit of 4. The three models are (see Fig. 1):

1. **Classical benchmark (linear / “triangle” model):**
  $$ E_c(\theta ) = -1 + {\frac{2\theta }{\pi }\qquad (0\le \theta \le \pi ).} $$
  With the extension to other angles understood by the usual symmetries, this is a standard rotationally covariant local hidden-variable correlation satisfying the singlet-like endpoint conditions $E(0)=-1$ and $E(\pi )=+1$ and providing a simple classical benchmark for angle-induced degradation. As shown in Sec. 5.2, Bell’s original inequality implies that within the restricted local class the small-angle behavior is necessarily at best linear-cusp-like, and the triangle model saturates the resulting bounds. For the conventional CHSH choice of settings used in this work, it attains the local bound $\mathcal {S}_{\textrm{CHSH}}^{(c)}=2$.
2. **Quantum (cosine):**
  $$ E_q(\theta ) = -\cos \theta . $$
  This describes measurements on a two-qubit singlet state and reaches the Tsirelson bound, $\mathcal {S}_{\textrm{CHSH}}^{(q)} = 2\sqrt{2}$.
3. **Stronger-than-quantum (step):**
  $$ E_s(\theta )= {\left\{ \begin{array}{ll} -1, &  0\le \theta<\pi /2,\\ 0, &  \theta =\pi /2,\\ +1, &  \pi /2<\theta \le \pi . \end{array}\right. } $$
  This hypothetical box correlation saturates the algebraic limit, $\mathcal {S}_{\textrm{CHSH}}^{(s)} = 4$ [22, 23]. The standard Popescu–Rohrlich box is defined for finitely many settings; the extension to a continuous function $E(\theta )$ is an additional idealization consistent with no-signaling but not uniquely fixed by the PR box definition.

## Thermodynamic value and robustness

### Peak work and mutual information

**Fig. 2:**
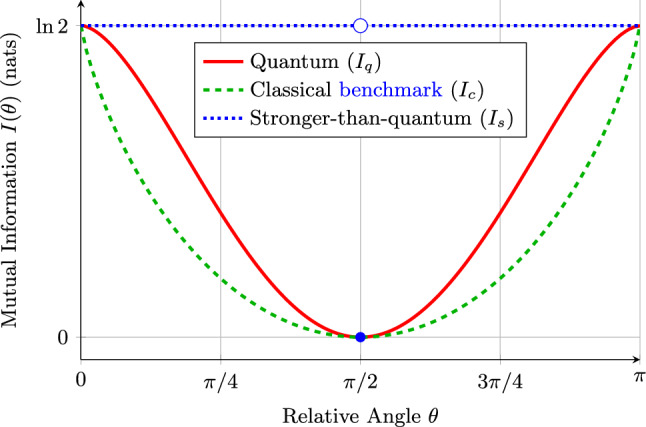
Mutual information versus measurement angle $\theta$. All three representative models achieve the maximum value $\ln 2$, but their robustness to misalignment differs. The classical benchmark (dashed green) degrades as $\Theta (\delta \theta \ln (1/\delta \theta ))$ from the peak, while the quantum cosine law (solid red) degrades as $\Theta (\delta \theta ^{2}\ln (1/\delta \theta ))$, offering superior resilience within the restricted comparison class

The thermodynamic value of these correlations is governed by the mutual information $I_\theta (A{:}E)$ derived in Eq. (7). As shown in Fig. 2, perfect correlation or anti-correlation ($|E(\theta )|=1$) yields the maximal mutual information $I=\ln 2$. Consequently, according to Eq. (3), the maximum work extractable per bipartite system in this binary-outcome, uniform-marginal setting is bounded by $k_{\textrm{B}}T\ln 2$. This peak value is achievable in all three representative models, for instance, by aligning the measurement bases ($\theta =0$).

### Robustness analysis

This thermodynamic equivalence in peak yield belies a critical operational difference: robustness. The practical advantage of nonclassical correlations becomes apparent when considering misalignment from the optimal setting ($\theta =0$).

*Classical benchmark (linear) robustness.* For the classical benchmark, $E_c(\delta \theta ) = -1 + 2\delta \theta / \pi$ near $\theta = 0$. Setting $p = (1 + E_c)/2 = \delta \theta / \pi$, the binary entropy for small *p* behaves as $h_2(p) \approx -p \ln p + p + \mathcal {O}(p^2)$. Therefore,

12 $$\begin{aligned} I_c(\delta \theta ) = \ln 2 - h_2\!\left( \frac{\delta \theta }{\pi }\right) \approx \ln 2 - \frac{\delta \theta }{\pi } \ln \!\frac{\pi }{\delta \theta } - \frac{\delta \theta }{\pi } + \cdots . \end{aligned}$$

The leading correction is $\Theta (\delta \theta \ln (1/\delta \theta ))$, worse than linear: The mutual information degrades sharply for any nonzero misalignment. This makes the chosen classical benchmark brittle with respect to experimental noise.

*Why the local benchmark is generic within the restricted class.* The use of a linear classical benchmark is not arbitrary. For local hidden-variable models with perfect anti-correlation, Bell’s original inequality [18] implies

13 $$\begin{aligned} |E(\textbf{a},\textbf{b})-E(\textbf{a},\textbf{c})| \le 1+E(\textbf{b},\textbf{c}). \end{aligned}$$

For rotationally covariant coplanar correlations $E(\textbf{a},\textbf{b})=E(|\alpha -\beta |)$, choose settings with $|\alpha -\beta |=2\theta$ and $|\alpha -\gamma |=|\beta -\gamma |=\theta$. Then

14 $$\begin{aligned} |E(2\theta )-E(\theta )| \le 1+E(\theta ). \end{aligned}$$

Since the restricted comparison class assumes monotonic increase on $[0,\pi ]$, the left-hand side equals $E(2\theta )-E(\theta )$, and therefore,

15 $$\begin{aligned} 1+E(2\theta )\le 2\,[1+E(\theta )]. \end{aligned}$$

Now suppose that near $\theta =0$ one has $1+E(\theta )\sim c\,\theta ^n$ with $c>0$. Equation (15) then implies $2^n\le 2$; hence,

16 $$\begin{aligned} n\le 1. \end{aligned}$$

Thus, no local rotationally covariant singlet-like model in the present comparison class can approach $-1$ quadratically at the origin; its small-angle behavior is necessarily at best linear-cusp-like. The triangle model saturates this constraint.

The same inequality constrains the CHSH angle used later. Writing $\delta (\theta ):=1+E(\theta )$, Eq. (15) gives $\delta (\pi /2)\le 2\delta (\pi /4)$ and $\delta (\pi )\le 2\delta (\pi /2)$; hence,

$$ 2=\delta (\pi )\le 4\delta (\pi /4). $$

Therefore, $\delta (\pi /4)\ge 1/2$. Using the singlet symmetry $E(\pi -\theta )=-E(\theta )$, we have $E(\pi /2)=0$, and thus, $E(\pi /4)\le 0$, so

17 $$\begin{aligned} |E(\pi /4)| = -E(\pi /4) \le \frac{1}{2}. \end{aligned}$$

Within the restricted local comparison class,

18 $$\begin{aligned} \mathcal {S}_W^{\mathrm{(local)}} \le 4k_{\mathrm B}T\!\left[ \ln 2-h_2\!\left( \tfrac{1}{4}\right) \right] , \end{aligned}$$

and the triangle model saturates this bound.

*Quantum (cosine) robustness.* For the quantum model, $E_q(\delta \theta ) = -\cos \delta \theta \approx -1 + \delta \theta ^2/2$. Setting $p = (1 + E_q)/2 \approx \delta \theta ^2/4$, we have $h_2(p) \approx -p\ln p + p \approx \frac{\delta \theta ^2}{4} \ln \!\frac{4}{\delta \theta ^2} + \frac{\delta \theta ^2}{4}$ for small $\delta \theta$. More precisely, expanding Eq. (7) with $E_q = -\cos \theta$ we can write

19 $$\begin{aligned} I_q(\delta \theta ) = \ln 2 - h_2\!\left( \sin ^2\!\frac{\delta \theta }{2}\right) \approx \ln 2 - \Theta (\delta \theta ^2 \ln (1/\delta \theta )). \end{aligned}$$

The leading correction is quadratic up to logarithmic factors. The quantum correlation $E_q(\theta ) = -\cos \theta$ has a stationary point at $\theta = 0$, which is the fundamental reason for this improved robustness. Within the present comparison class, the quantum resource is significantly more resilient against small imperfections in establishing a shared reference frame.

*Stronger-than-quantum (step) robustness.* The stronger-than-quantum model provides the ultimate in robustness: For any $\delta \theta \ne \pi /2$, we have $|E_s(\delta \theta )| = 1$, and therefore, $I_s(\delta \theta ) = \ln 2$. Coordination of measurement settings is almost entirely unnecessary; the resource is maximal for all but a single critical angle.

*General criterion.* The scaling hierarchy above is not an accident of the three specific models: Once a correlation law is specified, the robustness of the mutual information near a peak follows from the leading nonzero term in the expansion of $1+E(\theta )$ around the extremum. If

$$ E(\theta ) = -1 + c\,|\theta |^n + o(|\theta |^n) \qquad (c>0,\; n>0), $$

then the binary entropy implies

20 $$\begin{aligned} \ln 2 - I(\theta ) = \Theta (|\theta |^n \ln (1/|\theta |)). \end{aligned}$$

In particular, a linear cusp produces $\Theta (\delta \theta \ln (1/\delta \theta ))$ degradation, whereas a stationary quadratic extremum produces $\Theta (\delta \theta ^2\ln (1/\delta \theta ))$ degradation. Bell’s original inequality shows that, within the restricted local singlet-like class considered here, one must have $n\le 1$. The quantum cosine law instead has $n=2$. Thus, the thermodynamic advantage of the quantum example in this paper is not tied to the singlet state alone, but to the presence of a smooth stationary extremum. Table 1 summarizes the key properties of the three representative models.

**Table 1:** Summary of the three representative correlation laws. $\mathcal {S}_{\textrm{CHSH}}$ is the CHSH value; $W_{\max }$ is the peak extractable work (in units of $k_{\textrm{B}}T\ln 2$); the “Degradation of *I*” column gives the leading magnitude of the correction to $I(\delta \theta ) = \ln 2 - \cdots$ for small misalignment $\delta \theta$

Class	$\mathcal {S}_{\textrm{CHSH}}$	$W_{\max }/k_{\textrm{B}}T\ln 2$	Degradation of *I* near $\theta {=}0$
Classical benchmark	2	1	$\Theta (\delta \theta \ln (1/\delta \theta ))$
Quantum	$2\sqrt{2}$	1	$\Theta (\delta \theta ^{2}\ln (1/\delta \theta ))$
Stronger-than-quantum	4	1	0 (perfectly robust)

## Multi-setting thermodynamic robustness

The operational hierarchy established above can be quantified by an aggregate robustness parameter that evaluates the total thermodynamic value across multiple measurement settings, as would arise in a protocol where the measurement angle varies across runs.

We adopt the standard CHSH measurement configuration—Alice’s settings $\alpha _1 = 0$, $\alpha _2 = \pi /2$ and Bob’s settings $\beta _1 = \pi /4$, $\beta _2 = -\pi /4$—which produces four setting pairs with relative angles $\theta _{11} = \pi /4$, $\theta _{12} = \pi /4$, $\theta _{21} = \pi /4$, $\theta _{22} = 3\pi /4$. For isotropic correlations satisfying $|E(\theta )| = |E(\pi -\theta )|$, one has $I(\pi /4) = I(3\pi /4)$, so the four terms all take the same value.

We define the aggregate thermodynamic robustness parameter

21 $$\begin{aligned} \mathcal {S}_W = k_{\textrm{B}}T\sum _{k=1}^{4} I(\theta _k) = k_{\textrm{B}}T\bigl [3\,I(\pi /4) + I(3\pi /4)\bigr ]. \end{aligned}$$

*Important remark.* Although $\mathcal {S}_W$ is evaluated at the CHSH measurement angles, it is *not* a Bell inequality: No local bound is derived or claimed from sign-sensitive CHSH algebra. The standard CHSH functional depends on the *signs* of the correlators through the combination $E + E + E - E$, which is what enables the separation between local and nonlocal theories. The mutual information $I(\theta )$ depends only on $|E(\theta )|$ and is therefore insensitive to the sign structure. We use $\mathcal {S}_W$ purely as an operational figure of merit quantifying how much total work can be extracted when measurement settings span the CHSH configuration.

For the restricted local comparison class, Eq. (17) already implies the bound (18). The triangle model saturates that local bound. We now evaluate $\mathcal {S}_W$ explicitly for each of the three representative models.

*Classical benchmark.* Using Eq. (7) with $E_c(\pi /4) = -1/2$ and $E_c(3\pi /4) = 1/2$, so that $|E| = 1/2$ in both cases, we obtain $I(\pi /4) = I(3\pi /4) = \ln 2 - h_2(1/4)$. Computing $h_2(1/4)$ explicitly:

22 $$\begin{aligned} h_2\left( \tfrac{1}{4}\right)&= -\tfrac{1}{4}\ln \tfrac{1}{4} -\tfrac{3}{4}\ln \tfrac{3}{4} \nonumber \\&= 2\ln 2 - \tfrac{3}{4}\ln 3 \approx 0.5623 \;\text {nats}. \end{aligned}$$

Therefore, $I(\pi /4) = \ln 2 - (2\ln 2 - \tfrac{3}{4}\ln 3) = \tfrac{3}{4}\ln 3 - \ln 2 \approx 0.1308$ nats, and

23 $$\begin{aligned} \mathcal {S}_W^{(c)} = 4 \times 0.1308\, k_{\textrm{B}}T \approx 0.523\, k_{\textrm{B}}T. \end{aligned}$$

*Quantum.* With $E_q(\pi /4) = -\cos (\pi /4) = -1/\sqrt{2}$ and $E_q(3\pi /4) = -\cos (3\pi /4) = 1/\sqrt{2}$, we similarly have $|E| = 1/\sqrt{2}$ and $I(\pi /4) = I(3\pi /4) = \ln 2 - h_2\!\bigl (\tfrac{1+1/\sqrt{2}}{2}\bigr )$, where $p = (1-1/\sqrt{2})/2 = \sin ^2(\pi /8) \approx 0.1464$. Computing $h_2(0.1464)$ directly:

24 $$\begin{aligned} h_2(0.1464)&= -0.1464\ln (0.1464) - 0.8536\ln (0.8536) \nonumber \\&\approx 0.2813 + 0.1352 \nonumber \\&\approx 0.4165~\text {nats}. \end{aligned}$$

Therefore, $I(\pi /4) \approx 0.6931--0.4165 = 0.2766$ nats, and

25 $$\begin{aligned} \mathcal {S}_W^{(q)} = 4 \times 0.2766\, k_{\textrm{B}}T \approx 1.107\, k_{\textrm{B}}T. \end{aligned}$$

*Stronger-than-quantum.* With $|E_s(\pi /4)| = |E_s(3\pi /4)| = 1$, we have $I(\pi /4) = I(3\pi /4) = \ln 2 \approx 0.6931$ nats. Therefore,

26 $$\begin{aligned} \mathcal {S}_W^{(s)} = 4\,k_{\textrm{B}}T\ln 2 \approx 2.773\, k_{\textrm{B}}T. \end{aligned}$$

These values exhibit the strict hierarchy

27 $$\begin{aligned} \mathcal {S}_W^{(c)}< \mathcal {S}_W^{(q)} < \mathcal {S}_W^{(s)}. \end{aligned}$$

For the three representative laws studied here, stronger nonlocality coincides with a larger aggregate thermodynamic value across diverse measurement settings.

## Extensions and limitations

### Relaxing the uniform-marginal assumption

The derivation of the mutual information in Eq. (7) relied on two structural assumptions: (i) binary outcomes and (ii) uniform marginals ($P(a) = P(e) = 1/2$). Assumption (ii) can be relaxed straightforwardly at the level of definitions. For a general binary no-signaling distribution with marginals $P(a{=}+1) = q_A$ and $P(e{=}+1) = q_E$, the mutual information is

28 $$\begin{aligned} I(A{:}E) = H(A) + H(E) - H(A,E), \end{aligned}$$

where all entropies are Shannon entropies of the respective distributions. With nonuniform marginals, however, the joint distribution is no longer determined by $E(\theta )$ alone: One additionally needs the marginals. The work bound (3) remains valid, but the simple formula (7) is replaced by an expression involving the full binary table.

For binary outcomes, one still has $I(A{:}E) \le \min \{H(A), H(E)\} \le \ln 2$, so the peak extractable work remains at most $k_{\textrm{B}}T\ln 2$. But the present robustness comparison is specific to the binary, uniform-marginal setting, where $I(\theta )$ is determined solely by $E(\theta )$. Extensions beyond that setting require additional assumptions about the full joint distribution and are not pursued here.

### Beyond binary outcomes

For *d*-outcome measurements, the joint distribution is no longer captured by a single scalar correlation function, and the conditional entropy involves the full $d\times d$ probability table. The extractable work is still bounded by $k_{\textrm{B}}T\, I(A{:}E)$, but the direct link to a single angular correlator is lost. Accordingly, the smoothness argument developed here does not transfer automatically to $d>2$. Extending the robustness analysis beyond the binary case is a natural direction for future work.

### Single-shot corrections

The work bound (3) is an asymptotic many-copy result. In the single-shot regime, the relevant quantities are suitable smooth min and max entropies and the corresponding generalized free energies; see, for example, Refs. [11, 12]. A single-shot treatment of the present protocol would therefore replace the Shannon/von Neumann quantities used here by appropriate one-shot entropic measures. Whether the same robustness hierarchy persists in that regime is a natural conjecture, but it is not established by the present analysis.

### Connection to device-independent protocols

An analogous robustness advantage of quantum (and hypothetical beyond quantum) correlations appears in device-independent quantum key distribution [24] and certified randomness expansion [25], where Bell inequality violations can certify security even when the devices are imperfect. The thermodynamic robustness identified here is qualitatively similar: Within the restricted comparison class, smoother correlations degrade more gracefully under misalignment.

## Discussion and relation to prior work

Our results complement and extend several strands of the literature on correlations as thermodynamic resources.

The foundational connection between correlations and work was established by Oppenheim et al. [9], who introduced the work deficit—the gap between globally and locally extractable work—as a measure of quantum correlations. While the work deficit quantifies how much correlation *is* present, the present analysis addresses a different question: how robust a given correlation is as a fuel. The two perspectives are complementary.

Zurek [8] connected quantum discord to the work extractable by Maxwell’s demon, showing that the quantumness of correlations has thermodynamic consequences. The present work extends this perspective beyond quantum mechanics to include an idealized no-signaling comparison point, and it identifies robustness—rather than the absolute amount of extractable work—as the distinguishing feature among the three representative laws considered here.

Perarnau-Llobet et al. [26] provided a comprehensive framework for extractable work from general correlated states, proving tight bounds for local and global extraction protocols. The present work specializes to a Bell experiment-inspired scenario and reveals a hierarchy in robustness that is not visible in statements based solely on the total amount of correlation.

Funo et al. [27] showed that entanglement can provide a thermodynamic advantage in work extraction under global operations, while Hovhannisyan et al. [28] demonstrated that entanglement generation is not *necessary* for optimal work extraction by local operations. The finding that $W_{\max } = k_{\textrm{B}}T\ln 2$ for all three representative laws is consistent with the latter observation: In the present one-bit setting, the peak gross work is limited by the communicated information, not by nonlocality itself. The advantage of nonlocality here lies instead in the robustness with which that peak can be approached.

Gallego et al. [29] studied operational advantages of nonlocal correlations in information-theoretic tasks. The present work provides a thermodynamic analog: Within the restricted comparison class, the operational advantage is not a higher peak yield but a greater tolerance to misalignment.

The resource-theoretic approach to thermodynamics [10–12] introduces families of generalized free energies and establishes second laws beyond the standard one. The bound in Eq. (3) is the asymptotic many-copy version of these constraints. Single-shot corrections would alter the quantitative values of $\mathcal {S}_W$, but their effect on the present robustness hierarchy has not been analyzed here.

It is important to clarify the scope of these results. The bound in Eq. (3) is standard within information thermodynamics: For systems coupled to a heat bath at temperature *T*, under the usual assumptions of the generalized second law, the maximum average work extractable in a cyclic protocol is bounded by $k_{\textrm{B}}T$ times the appropriate mutual information. In the operational setting studied here this is applied to the classical outcome registers generated by the parties’ measurements. The expression for $I_\theta$ in Eq. (7) is similarly model-independent: It follows solely from binary outcomes, uniform marginals and the correlator $E(\theta )=\langle AE\rangle _\theta$.

What is model-dependent is the specific form of $E(\theta )$. The linear function is a standard rotationally covariant local model and, within the restricted singlet-like local class adopted here, Bell’s original inequality forces the small-angle behavior to be at best linear-cusp-like and the CHSH angle value to satisfy $|E(\pi /4)|\le 1/2$; the triangle model saturates those bounds. The cosine law arises from the singlet state, and the step law is an idealized continuous no-signaling form reaching $\mathcal {S}_{\textrm{CHSH}}=4$ [23]. The robustness comparison should therefore be read as a statement about this restricted comparison class, not as a classification of all correlations permitted by a given theory.

## Conclusion

Correlations between a system and its environment are a thermodynamic fuel whose value in the present binary-outcome, uniform-marginal setting is limited by their mutual information. A single perfectly correlated bit can yield at most $k_{\textrm{B}}T\ln 2$ of gross work, as exemplified by a Szilard engine powered by shared information [4]. Engines consuming correlations do not violate the second law; they demonstrate that information must be treated as a physical resource [5, 6].

Within the restricted comparison class of representative singlet-like correlation laws studied here, the hierarchy among classical, quantum and stronger-than-quantum correlations lies not in the peak value but in the robustness with which it can be accessed. The local singlet-like class is constrained by Bell’s original inequality to be at best linear-cusp-like near perfect alignment, whereas the quantum cosine law has a smooth stationary extremum and is therefore more resilient to small misalignment. The idealized no-signaling step law is flatter still. In this sense, quantum correlations are more robust than the classical benchmark: not because they raise the peak gross yield in the present one-bit setting, but because they preserve that yield more effectively under misalignment. The idealized no-signaling step law is more robust still.

The robustness hierarchy identified here should therefore be read as a statement about a restricted comparison class of singlet-like, rotationally covariant correlations, not as a theorem covering all possible classical shared randomness strategies. Within that class, the quantum cosine law is markedly more tolerant to misalignment than the classical linear benchmark, while the idealized no-signaling step law is flatter still.

## Data Availability

Data sharing not applicable to this article as no datasets were generated or analyzed during the current study.
